# Glycemic control associated factors in type 1 diabetes mellitus patients: a cross sectional study

**DOI:** 10.1186/1758-5996-7-S1-A46

**Published:** 2015-11-11

**Authors:** Patrícia Ramos Guzatti, Amely PS Balthazar, Maria Heloisa Busi da Silva Canalli, Thais Fagnani Machado

**Affiliations:** 1 Unisul Pedra Branca, Florianópolis, Brazil

## Background

Type 1 diabetes mellitus is one of the leading chronic diseases in children and represents 5 to 10% of all diabetes cases. The International Diabetes Federation's atlas described T1D's incidence in people under 15 yrs. old in the world: the major incidence was found in Finland with 57.6 new cases in 100,000 inhabitants. In Brazil, the incidence is 10.4 cases in 100,000 inhabitants. Intensive treatment, by lowering HbA1c, is effective in reducing the onset and progression of diabetic's chronic complications. In addition, physical activity, diet based on carbohydrate counting and education about the illness are also measures to improve glycemic control.

## Objective

Compare sociodemographic factors, physical activity practice, treatment related factors and chronic complications with HbA1c in type 1 diabetics.

## Materials and methods

A cross-sectional study conducted in the Endocrinology Ambulatory of Governador Celso Ramos Hospital, through a census of records, totaling 48 records. Patients from 14 to 50 yrs. old who had been seen at least twice were included. The illiterate patients, those with renal terminal disease and with hemoglobin <10g/dl were excluded. Glycemic control was evaluated according to sociodemographic factors, lifestyle, factors related to treatment, in accordance with the Brazilian Diabetes Society Guideline. The descriptive and bivariate analyzes by Fisher's exact test showed a significance level of 95% (p≤0.05). Approved by HGCR ethics committee.

## Results

A prevalence of men (54.17%), with < 25 yrs. of age (56.25%) and more than ten yrs. of disease (52.08%) was observed. The overall HbA1c mean was 8.68% (±2.17); and values ≤ 7% were obtained in 22.92% of the population. Among those with adequate glycemic control, 90.91% lived in Greater Florianópolis, 60% engaged in physical activity, 63.64% used insulin analogues, 90.91% measure self-blood glucose three or more times/day and 100% applied insulin three or more times/day. Of patients with HbA1c ≤ 7, 100% had no chronic complications.

## Conclusion

Practicing physical activity, conducting intensive insulin therapy, using insulin analogues and be a resident of Greater Florianópolis were the main factors that contributed to the adequate glycemic control. One of the possible actions to be taken based on this study, with the aim of improving glycemic control, would be the implementation of a multidisciplinary team in the Endocrinology Ambulatory, in order to promote educational activities.

